# The Mental Health Impact of Daily News Exposure During the COVID-19 Pandemic: Ecological Momentary Assessment Study

**DOI:** 10.2196/36966

**Published:** 2022-05-25

**Authors:** John K Kellerman, Jessica L Hamilton, Edward A Selby, Evan M Kleiman

**Affiliations:** 1 Department of Psychology Rutgers, The State University of New Jersey Piscataway, NJ United States

**Keywords:** news consumption, worry, hopelessness, ecological momentary assessment, news media, COVID-19, pandemic, mental health, depression, stress, psychological distress, mediation model, digital health

## Abstract

**Background:**

Consumption of distressing news media, which substantially increased during the COVID-19 pandemic, has demonstrable negative effects on mental health.

**Objective:**

This study examines the proximal impact of daily exposure to news about COVID-19 on mental health in the first year of the pandemic.

**Methods:**

A sample of 546 college students completed daily ecological momentary assessments (EMAs) for 8 weeks, measuring exposure to news about COVID-19, worry and optimism specifically related to COVID-19, hopelessness, and general worry.

**Results:**

Participants completed >80,000 surveys. Multilevel mediation models indicated that greater daily exposure to news about COVID-19 is associated with higher same-day and next-day worry about the pandemic. Elevations in worry specifically about COVID-19 were in turn associated with greater next-day hopelessness and general worry. Optimism about COVID-19 mediated the relationship between daily exposure to COVID-19 news and next-day general worry but was not related to hopelessness.

**Conclusions:**

This study demonstrates the mental health impact of daily exposure to COVID-19 news and highlights how worry about the pandemic contributes over time to hopelessness and general worry.

## Introduction

### Background

The uncertain and rapidly changing nature of the pandemic, coupled with shelter-in-place and work-from-home orders in the first year of the pandemic, increased access to and demand for information about COVID-19 [1,2]. News consumption increased across multiple forms of media, including increases in television news viewership, daily visits to news websites, and the use of mobile phones to monitor pandemic updates [3-5]. The increased consumption of negative news during the pandemic likely has a negative impact on mental health [6-9]. This study examines 2 questions about the short- and long-term mental health consequences of engaging with news media about COVID-19: (1) What is the short-term relationship between daily news exposure and distress about the pandemic? (2) How does COVID-19-specific distress generalize over time to broader effects on mental health?

### Media Exposure and Mental Health

Crisis situations prompt information seeking to reduce uncertainty and increase feelings of safety, corresponding to an increase in overall news consumption during the pandemic [2,8,10,11]. During periods of elevated stress, people may turn to media outlets to help relax and cope with anxiety, particularly when access to other coping resources is limited [12-15]. However, naturalistic and experimental research has found a relationship between watching distressing news and lower emotional well-being, including elevated anxiety and worry [16-20]. Evidence indicates that elevated exposure to news coverage of mass trauma events (eg, natural disasters, terrorist attacks) predicts symptoms of anxiety, depression, and posttraumatic stress, even for individuals not directly exposed to the traumatic event [16,19,21,22]. These effects persist across multiple mediums, including news consumed via print, radio, television, and social media posts. Secondary sources (eg, social media) are increasingly relied upon for news updates, and young adults reported higher daily engagement with news via social media than any other source early in the pandemic [23,24]. Even brief exposure to negatively valenced news content (eg, watching 15-minute video clips) increases anxious and sad mood immediately following exposure [25]. Szabo and Hopkinson [9] found that changes in mood persisted after participants engaged in a distracting task following exposure, suggesting that the affective impact of news consumption extends beyond momentary reactions.

### News About COVID-19 and Mental Health

A nationally representative, cross-sectional survey found that consuming news about COVID-19 was associated with greater psychological distress in the week following the implementation of stay-at-home orders in the United States [8]. Individuals who reported following the news “very closely” reported the highest levels of distress, which were associated with increases in perceived threat of the virus [8,11,26]. Greater frequency and duration of engagement with news about COVID-19 are associated with symptoms of anxiety and depression regardless of source, with individuals who consume COVID-19-related media across multiple platforms reporting the greatest increases in symptom severity [7,27]. A recent systematic review indicated that increased consumption of news about the pandemic was broadly associated with mental health decline among young people in international samples [28]. However, there has been limited examination of specific, proximal effects of news consumption on daily mental health and distress.

In addition to intentional engagement, exposure to COVID-19-related news may be incidental. Due to evolutions in news dissemination across social network platforms, millions are inadvertently exposed to distressing content while engaging with social media for connection, social support, and distraction, limiting the efficacy of web-based coping methods and increasing worry [29-32]. Distress may be further compounded in the context of COVID-19 because of an increase in misinformation and uncertainty about news accuracy, which may prompt additional news engagement in the light of rapidly changing and contradictory information presented by various sources [2,33,34].

### Worry and Optimism About COVID-19

COVID-19 represents an ongoing crisis for which much of the coverage has centered on current and future threats [8]. Given the corresponding uncertainty across multiple domains of life, worry and optimism about COVID-19 may be particularly relevant for broader mental health over time. As new variants emerge and the effects of COVID-19 continue to unfold worldwide, increased consumption of news media about COVID-19 may contribute to higher levels of worry and lower optimism about the pandemic [35].

The proximal mental health impact of daily exposure to news about COVID-19 may generalize over time. Cross-sectional research indicates that greater exposure to COVID-19 media across various platforms is associated with higher levels of both general anxiety and anxiety specifically about COVID-19, although the relationship between the 2 forms of anxiety is not yet understood [27]. Engaging with COVID-19 news may lead to worry specifically about COVID-19, which then generalizes over time. Previous work has found that worry prompted by news consumption generalizes to worried cognitive patterns beyond the topics covered in the distressing media [25]. Brief exposure to negative television news enhances the tendency to “catastrophize” unrelated personal worries, increasing expectations of worst-case outcomes for personal future events. However, studies that have tested the speculated generalization of worry following news consumption have been cross-sectional and thus not able to test this mediational chain in a way that intensive longitudinal data allows.

Lower levels of optimism have been found to predict greater psychological distress among adults during COVID-19, and the previous literature indicates that higher levels of pessimism are related to a broad range of mental and physiological difficulties [36-38]. Further, lower levels of optimism mediate the relationship between news exposure and psychological distress [18]. Much of the existing research on the mental health outcomes associated with optimism have examined optimism and pessimism cross-sectionally or as trait-level constructs rather than examining the predictors and antecedents of short-term fluctuations in optimism [36,39,40].

Engaging with news that decreases optimism about COVID-19 may contribute to broader psychiatric symptoms, including worry and hopelessness. International research has found that hopelessness, which is associated with lower optimism about the future [41,42], has increased among adult samples during the pandemic [43]. News outlets and mental health experts have speculated that consumption of media related to COVID-19 may increase hopelessness [44-46]. However, this assertion has not been empirically evaluated, nor has the relationship between optimism specifically about COVID-19 and broader hopelessness. Importantly, the proximal, day-to-day mental health effects of worry and optimism about COVID-19 have not yet been assessed.

### This Research

This research is among the first to use real-time monitoring within the context of COVID-19 and, to the best of our knowledge, the only paper to examine the impact of daily exposure to news about COVID-19 on mental health among undergraduate students. Undergraduate students may be particularly vulnerable to the mental health impact posed by the pandemic, given displacement due to campus closures, reduced access to in-person social networks, and uncertainty about graduation and future employment opportunities [29,47,48]. Given the ongoing nature of the pandemic, fluctuations in daily news consumption, and the prevalence of inadvertent exposure, it is crucial to understand the proximal, rapidly changing mental health impact of news about COVID-19 in ways that have not been captured in existing cross-sectional research on media consumption and mental health. Accordingly, in this study, we utilized intensive longitudinal data collection (ie, ecological momentary assessment [EMA]) to capture these rapid changes.

The primary research questions, context, and hypotheses were as follows:

- What is the impact of daily exposure to news about COVID-19 on mental health? Daily levels of worry and optimism about COVID-19 were assessed over an 8-week study period in the early months of the pandemic. Previous research conducted with this sample indicates that COVID-19 worry and anxiety are significantly higher than general, nonspecific anxious feelings among undergraduates [49]. Given previous research [35], we hypothesized that greater daily exposure to news about COVID-19 would lead to increased worry and decreased optimism about the pandemic.
- What are mechanisms through which exposure to news about COVID-19 increases mental health issues? Momentary levels of hopelessness and general worry were examined over the same 8-week period. Previous findings indicate that specific worry can generalize following news consumption [25] and that optimism/pessimism mediates the relationship between news exposure and psychological distress [18]. Building upon our first hypothesis, we expected that greater daily exposure to news about COVID-19 would lead to increased worry and decreased optimism about the pandemic, which would then generalize to general worry and hopelessness.

## Methods

### Participants and Procedure

Data (N=546) were drawn from an ongoing study assessing stress, emotion, and behavior among a general college student sample. Recruitment took place between April and December 2020 following the initiation of stay-at-home orders. Participants were remotely recruited via the undergraduate psychology research pool and introductory courses at a large, public university. Recruitment was open to all students who met eligibility criteria, which were assessed using an online screener. Eligible individuals were aged 18+ years, had access to a smartphone compatible with EMA survey software (MetricWire), and were willing to complete multiple surveys per day across the study period. Upon completion of an informed consent protocol, eligible participants were asked to complete an online baseline assessment and were provided with instructions for downloading MetricWire, a smartphone app used to deliver EMA surveys for the 8-week period. Compensation was provided for completing the baseline assessment and for each of the EMA surveys completed during the study period, with a possible total of US $141 for completing all study procedures. For further recruitment and compensation details, see Kleiman et al [49].

### EMA Surveys

Participants completed 6 EMA surveys daily for 8 weeks; 5 surveys were delivered at random times throughout the day to capture a range of real-time responses. The surveys were brief (under 5 minutes) and asked participants to report their in-the-moment feelings (ie, “Right now, how much do you feel”) across a range of affect states (eg, excited, sad) on a 0 (*not at all*) to 10 (*very much*) scale. To capture in-the-moment affect, including hopelessness and general worry, single items adapted from the Positive and Negative Affect Schedule (PANAS [50]) were used in each momentary survey. Each EMA survey included a question about COVID-19 worry (ie, “How worried or anxious are you about the coronavirus outbreak?”), with answers ranging from 0 (*not at all*) to 5 (*very much*).

The sixth and final survey delivered each night included items asking participants to reflect back across the day. Nightly surveys included a single-item assessment of optimism about COVID-19 (ie, “What best describes how optimistic you feel about the coronavirus outbreak?”), rated on a 0 (*very pessimistic*) to 5 (*very optimistic*) scale. Finally, the nightly survey asked participants to share the frequency with which they were exposed to news about COVID-19 across the day (ie, “How frequently did you see or read news or media about coronavirus today?”) on a scale from 0 (*not at all*) to 5 (*very much*). Questions related to COVID-19 were asked using single items developed for this study.

### Analytic Strategy

Across the sample, participants submitted a total of 86,626 survey responses. Individual surveys were excluded from analyses if there was no variability across responses to all items in that specific survey (SD 0 across all variables scored on a Likert scale), which removed 726 (0.8%) survey responses. Since optimism about the coronavirus outbreak and exposure to COVID-19 news were assessed once per day, momentary variables of interest (ie, hopelessness, general worry, and worry about COVID-19) were aggregated to create daily average scores for each variable for each participant. Participants who recorded <3 days of EMA data were excluded from analyses, yielding a final sample of 19,888 days of data across 546 participants.

Given the hierarchical structure of our data, multilevel mediation analyses were used to test our hypothesis that COVID-19 worry and optimism would mediate the relationship between COVID-19 news exposure and general worry and hopelessness. We aggregated momentary variables to create a daily average for each participant, resulting in a nested structure where daily observations (level 1) were nested within people (level 2). Mediator and outcome variables were examined using intraclass correlation (ICC) to determine the proportion of variance attributable to within-person and between-person differences across the study period. We person-mean-centered all exogenous variables.

**Figure 1:**
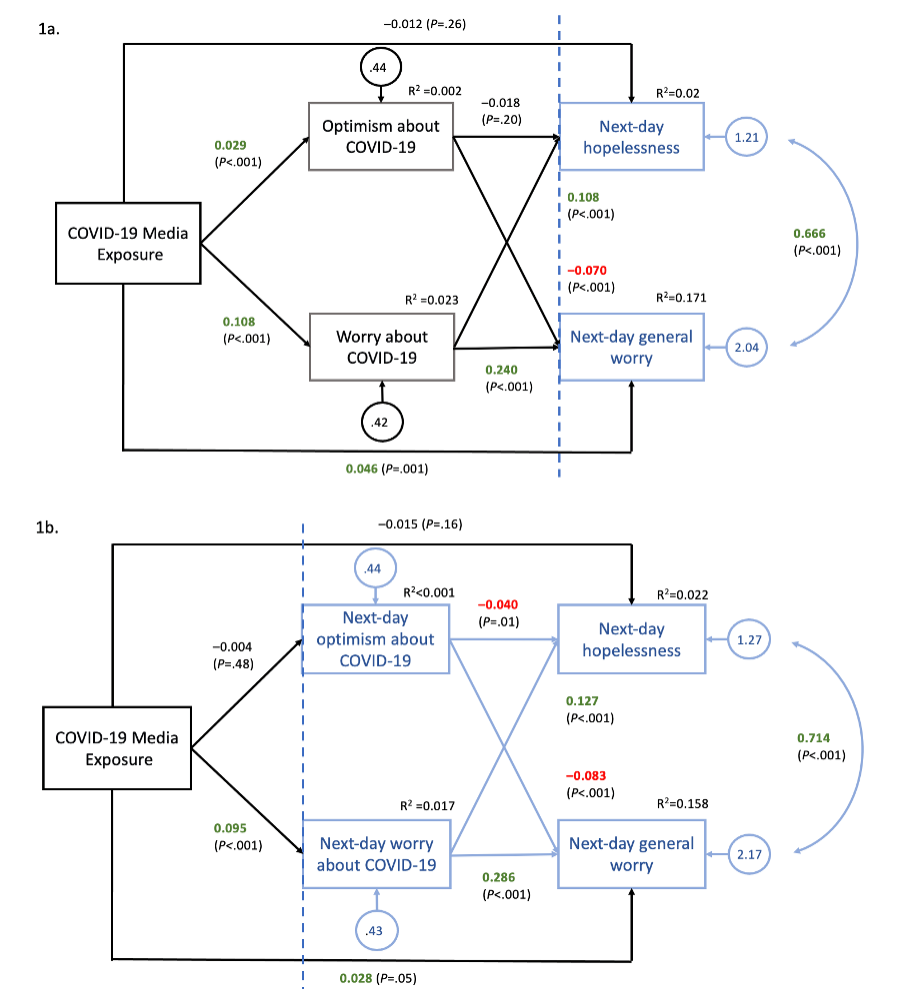
Temporal multilevel mediation models with next-day effects. Note: the vertical dashed line indicates the break between consecutive days of data (ie, boxes shown in the same color represent data collected on the same day). Models show the effect of variables at time T (outlined in black) on variables the following day (T+1; outlined in blue). Coefficients in green indicate a positive relationship; coefficients in red indicate a negative relationship.

We conducted 2 multilevel mediation analyses with frequency of exposure to COVID-19 news entered as the predictor, COVID-19 worry and optimism as mediators, and hopelessness and general worry as outcome variables (see Figures 1a and 1b). The predictor, mediator, and outcome variables were each assessed daily across the study period. Therefore, all variables were entered at the same level (observation), creating a 1-1-1 (ie, predictor-mediator-outcome) multilevel mediation model. Temporal models used repeated-assessment data to examine next-day effects of COVID-19 news exposure. In the first model, the predictor and mediator variables were lagged to predict next-day outcomes (ie, COVID-19 media exposure, worry, and optimism at time T predicted hopelessness and general worry at time T+1; see Figure 1a). In the second model only the predictor variable was lagged to predict next-day mediator and outcome variable scores (ie, COVID-19 media exposure at time T predicted COVID-19 worry, COVID-19 optimism, hopelessness, and general worry at time T+1; see Figure 1b). Temporal analyses were restricted to consecutive-day pairs. Model fit was evaluated using criteria recommended by Hu and Bentler [51]: root-mean-square error of approximation (RMSEA)≤0.06 and comparative fit index (CFI)≥0.95 were considered cut-offs for adequate model fit.

To account for individual differences, a supplemental model was run with person means for all variables included at level 2 (see Multimedia Appendix 1). To examine the relationship between the predictor variables and next-day worst-point outcomes, a second exploratory model used the highest score endorsed for hopelessness and general worry by each participant on a given day rather than daily participant averages (see Multimedia Appendix 2). The 2 additional models yielded similar results as the original models and are thus included as Multimedia Appendices 1 and 2.

Effect sizes for the direct effects of COVID-19 media exposure on the endogenous variables were calculated using R^2^. As recommended by Preacher and Selig [52], 95% Monte Carlo CIs were used to examine indirect effects. All analyses were conducted using R (R Core Team) with the lavaan, semTools, and EMAtools packages [53-56].

### Ethical Considerations

This study was approved by the Rutgers University Institutional Review Board (IRB #: Pro2019002249).

## Results

### Participant Details

Between April 24, 2020, and January 31, 2021, our final sample of 546 undergraduate students completed 80,779 assessments over a total of 19,888 days of data across participants (see Table 1 for a summary of the survey schedule and responses). On average, participants gave at least 1 survey response on a total of 36.4 days across the 8-week study period for an overall response rate of 67.7% across those days. This study used a longer EMA study period (8 weeks) than most previous EMA research conducted with college students. On average, there was a slight reduction in the response rate between the participants’ first 4 weeks of the EMA period (response rate=69.4%) and the last 4 weeks (response rate=65.1%). The participants’ average age was 19.44 years (SD 1.99, range 18.01-33.23), and 131 (24.2%) were first-generation college students. Further demographic information is presented in Table 2.

Table 3 shows descriptive statistics and ICCs for all variables, with ICCs indicating noteworthy between-person to within-person variance. Parameter estimates for both mediation models are shown in Figure 1a and Figure 1b, respectively. Across multiple fit indices, both the first (X^2^_(7,N=533)_=225.02, *P*<.001, CFI=0.95, RMSEA=0.047) and second (X^2^_(7,N=538)_=198.86, *P*<.001, CFI=0.95, RMSEA=0.044) models demonstrated good model fit.

Hypothesis 1 was partially supported by examining the direct effects of changes in the frequency of daily COVID-19 media exposure on worry and optimism about the pandemic. In the first model (see Figure 1a), greater news exposure was significantly associated with greater same-day worry about COVID-19 (*P*<.001). Counter to our hypothesis, a higher frequency of news exposure on a given day was also significantly associated with higher levels of same-day optimism about COVID-19 (*P*<.001). Similar associations were found in the second model (see Figure 1b) for the relationship between news exposure and next-day COVID-19 worry (*P*<.001), but no relationship was found between frequency of news exposure and next-day optimism about COVID-19 (*P*=.48).

For hypothesis 2, we first examined the direct relationship between COVID-19 news exposure and general worry and hopelessness. In the first model, a significant direct effect was found between increased news exposure and increased next-day general worry (*P*=.001) but there was no significant direct effect of media exposure on next-day hopelessness (*P*=.26). Similar results were found for the second model such that news exposure exhibited a significant, albeit close to *P*=.05, direct effect on next-day general worry (*P*=.047) but not on next-day hopelessness (*P*=.16) when accounting for next-day specific worry and optimism.

COVID-19 worry was significantly associated with same-day general worry (*P*<.001) and same-day hopelessness (*P*<.001). Similar results were found for the relationship between COVID-19 worry and next-day general worry (*P*<.001) and hopelessness (*P*<.001). COVID-19 optimism was significantly associated with same-day general worry (*P*<.001) and same-day hopelessness (*P=*.01)*.* COVID-19 optimism was also associated with lower next-day general worry (*P*<.001), but no significant relationship was found between COVID-19 optimism and next-day hopelessness.

We then examined the indirect effects of COVID-19 news exposure on hopelessness and general worry, as mediated by COVID-19 worry and optimism. Significant indirect effects were found such that worry about COVID-19 mediated the relationship between same-day COVID-19 news exposure and both next-day general worry (b=0.026, 95% CI 0.021-0.031) and next-day hopelessness (b=0.012, 95% CI 0.008-0.015). Optimism about COVID-19 also mediated the relationship between same-day COVID-19 news exposure and next day general worry (b=–0.02, 95% CI –0.003 to –0.001), but no significant mediation effect was found for the relationship between news exposure and next-day hopelessness. Similar significant indirect effects were found in the second model such that COVID-19 worry also mediated the relationship between prior-day news exposure and same-day general worry (b=0.027, 95% CI 0.022-0.032) and same-day hopelessness (b=0.012, 95% CI 0.009-0.015). Optimism about COVID-19 did not mediate the relationship between news exposure and same-day outcomes.

Additional models yielded similar results as the original temporal models. Including person means and examining worst-point daily outcomes rather than daily averages did not meaningfully change the findings (see online Multimedia Appendices 1 and 2 for model details).

**Table 1:** Schedule and number of responses for each assessment.

Level	Schedule	Variables assessed at this level	Number of responses included in analyses, n (%)
Person	1x at the beginning of the study period	Baseline measures (demographic information)	546 (100)
Daily	1x every night for 8 weeks	Frequency of exposure to news about COVID-19, optimism about COVID-19	31,997 (39.6)
Momentary	5 random times daily for 8 weeks	Hopelessness, general worry, worry about COVID-19	48,802 (60.4)

**Table 2:** Participants’ (N=546) sociodemographic variables.

Characteristics		Participants, n (%)
**Gender**		
	Women	398 (72.9)
	Men	139 (25.5)
	Nonbinary/gender expansive	8 (1.47)
	Not reported	1 (0.02)
**Racial background**		
	White	192 (34.04)
	Asian	268 (49.08)
	Black or African American	31 (5.68)
	Bi-/multiracial	21 (3.84)
	Middle Eastern	7 (1.28)
	American Indian or Alaska Native	7 (1.28)
	Other racial background	15 (2.75)
	Not reported	3 (0.55)
**Ethnicity**		
	Non-Hispanic or non-Latino/non-Latina	479 (87.73)
	Hispanic or Latino/Latina	67 (12.27)
**Annual household income (US $)**		
	<10,000 to <30,000	30 (5.49)
	30,000 to <50,000	48 (8.79)
	50,000 to <70,000	34 (6.23)
	70,000 to <100,000	63 (11.54)
	100,000 to <200,000	171 (31.32)
	≥200,000	43 (7.88)
	Not reported	157 (28.75)

**Table 3:** Mean (SD) and ICC^a^ for all variables across the study period.

Variable	Mean (SD)	ICC (95% CI)
How frequently did you see or read news or media about coronavirus today?	1.53 (1.5)	0.62 (0.59-0.65)
How worried or anxious are you about the coronavirus outbreak?	2.49 (1.66)	0.83 (0.81-0.85)
What best describes how optimistic you feel about the coronavirus outbreak?	2.49 (1.19)	0.69 (0.66-0.72)
Right now, how much worried do you feel?	2.69 (2.77)	0.69 (0.66-0.71)
Right now, how much hopeless do you feel?	1.62 (2.38)	0.74 (0.71-0.76)

^a^ICC: intraclass correlation.

## Discussion

### Principal Findings

We used real-time monitoring to quantify the short- and long-term mental health impact of daily exposure to news about COVID-19 over time. We aimed to answer 2 primary questions: (1) Does greater daily exposure to news media about COVID-19 predict elevations in same-day and next-day worry and pessimism specifically about the pandemic? (2) Do COVID-19 worry and optimism generalize to broader worry and hopelessness?

### Mental Health Impact of Exposure to News About COVID-19

Our first hypothesis was partially supported. Greater daily exposure to news about COVID-19 was associated with elevated same-day and next-day COVID-19 worry. However, greater exposure to COVID-19 news was also associated with elevated same-day optimism about the pandemic, suggesting that engaging with information about COVID-19 may have both proximal beneficial and deleterious effects on mental health. No relationship was found between frequency of media exposure and next-day optimism about the pandemic. Our second hypothesis that COVID-19 worry and optimism would mediate the relationship between COVID-19 media exposure and general worry and hopelessness was also partially supported. In both temporal models, mediation effects were found such that greater COVID-19 worry mediated the relationship between increased news exposure and general worry as well as the relationship between increased news exposure and hopelessness. Both models also yielded significant direct effects between increased news exposure and increased general worry, indicating that consuming more news about the pandemic increases nonspecific worry beyond what is accounted for by the mediation effects of COVID-19 worry.

These findings add to a limited body of literature suggesting that specific worry prompted by distressing media generalizes to more diffuse worry [25]. Greater exposure to COVID-19 news is associated with increases in COVID-19 worry on the same and the next day. Elevated specific worry predicts next-day general worry, indicating a generalization effect through which specific worry broadens over time. This suggests that the harmful effects of distressing news about the pandemic persist rather than being limited to momentary reactions during and immediately following exposure. Worry about COVID-19 is not inherently harmful and can increase preventive behavior to reduce virus transmission [11]. However, given the ubiquity of COVID-19 media and distressing media beyond the pandemic, the generalization of COVID-19 worry to broader anxious symptoms and feelings of hopelessness may contribute to poor mental health outcomes.

### Optimism About COVID-19 and Hopelessness

Optimism about COVID-19 was significantly associated with decreased same-day hopelessness and decreased same-day and next-day general worry. COVID-19 optimism also mediated the relationship between increased news exposure and next-day general worry, suggesting that the protective effects of some news consumption may extend beyond same-day reactions. Across our sample, participants were moderately optimistic about COVID-19, reporting higher daily averages for optimism than all other variables examined. This suggests that college students may maintain a general sense of optimism about the pandemic independent from worry, hopelessness, and media exposure. The observed within-person variability in COVID-19 optimism scores (ICC=0.69) supports the examination of optimism about the pandemic as a state-level variable prone to changes over short periods of hours and days.

There was mixed support for the relationship between COVID-19 news consumption and hopelessness. There was no direct effect of COVID-19 news exposure on same-day or next-day hopelessness. Indirect effects, however, were found in both models through COVID-19 worry, indicating that COVID-19 media consumption contributes to hopelessness by increasing COVID-19 worry. Given the uncertainty and limited information about the virus in 2020, perceived helplessness and lack of control may have accompanied worry about COVID-19 and contributed to hopeless feelings. More research is needed to determine the nature of the association between specific worry and hopelessness and to identify other possible mediating factors driving the relationship between hopelessness and exposure to distressing media.

### Implications

This study highlights the potential risks of increased media consumption during the COVID-19 pandemic. Although information-seeking behavior during a crisis may be intended to reduce uncertainty and distressing emotions, these findings suggest that regular exposure to COVID-related media may instead lead to deleterious mental health outcomes by increasing worry about the pandemic, which in turn increases general worry and hopelessness. Daily engagement with news may represent a modifiable clinical target, and coping strategies that minimize exposure to distressing news may be beneficial during and beyond the pandemic.

This study also illuminates a potential benefit of COVID-19 news consumption, which increased same-day optimism about the pandemic, which in turn was associated with decreased next-day general worry. Parallel relationships between news exposure and COVID-19 worry and optimism imply that engaging with news about the pandemic contributes to multiple possible outcomes. It is possible that certain types of news stories increase optimism, while others increase worry. It is also possible that frequent news consumption is beneficial for some individuals and harmful for others. During a pandemic in which preventive behaviors (eg, socially distancing, wearing a mask, washing hands) can contribute to individual safety and decrease the spread of the pandemic, worry and optimism may each serve adaptive and maladaptive functions. Elevated worry may contribute to mental health difficulties but may also increase adaptive safety behaviors. Recent research has found that individuals who perceive COVID-19 as a high-risk threat are more likely to engage in protective behavior than individuals who are less worried about the personal risk of the pandemic [40]. Optimism may help buffer against depressive and anxiety symptoms during the pandemic, but COVID-19 optimism has been linked to lower engagement in protective behaviors and biased perceptions of risk [40,57]. Together, these findings suggest that engaging with news media about COVID-19 is not inherently harmful, nor is it necessarily beneficial.

News coverage and publicly available information about COVID-19 play an important role in perceptions of risk and subsequent preventive behaviors. Previous research has indicated that only specific types of media engagement (ie, news from official government or health organizations) are related to preventive behaviors but that COVID-19 news consumption increases anxiety and distress regardless of platform or media source [11,26,27]. These findings illuminate the importance of accurate and easily accessible news about the pandemic. Individuals who are informed about certain aspects of COVID-19 (eg, methods of transmission, efficacy of preventive measures) report higher optimism and lower anxiety than individuals who endorse inaccurate information about the pandemic [58]. Future research should examine which types of news and which mediums of information delivery are associated with adaptive behaviors and minimize the generalization and harmful effects of corresponding elevations in worry.

The generalization of specific worry highlights opportunities for brief interventions to buffer against the deleterious generalization effects of inadvertent news exposure. Szabo and Hopkinson [9] found that individuals who engaged in a brief (ie, 15-minute), progressive relaxation exercise after watching distressing television news experienced a more rapid return to affective baseline compared to participants who engaged in a distraction exercise. Prolonged increases in worry and hopelessness may contribute to the development or worsening of psychiatric disorders, including anxiety and mood disorders. Understanding the mechanisms through which exposure to news contributes to anxious and depressive symptoms as well as identifying opportunities to disrupt the generalization of worry are important targets to reduce the mental health impact of distressing news exposure.

### Limitations and Strengths

There were limitations of this study that should be acknowledged. We assessed daily news exposure using a broad self-report item in the nightly surveys, which limited examination of specific types of news engagement and assessment of when news consumption occurred during the day. COVID-19 optimism and news exposure were assessed retrospectively at the daily level rather than using momentary assessments. Previous findings from this sample indicate that participants report higher anxiety about COVID-19 on days when the number of new COVID-19 cases announced were higher in participants’ state of residence [49]. Future research using more comprehensive assessments of news engagement may provide additional information about the extent of individuals’ engagement with specific types of news. Recruitment was limited to students who were willing and able to complete multiple daily surveys over 8 weeks, and our sample may have systematic differences from other students (eg, less busy schedules), potentially reducing generalizability. All participants were undergraduate students recruited from the same university, likely further limiting generalizability to other populations. The identified relationships between COVID-19 news exposure and mental health should be studied in other populations, including clinical samples with higher levels of distress.

This study had several strengths. It is the largest and most fine-grained study to date on the relationship between frequency of daily news exposure and same-day and next-day mental health outcomes among college students. This study used consecutive days of data collection to test the influence of variables specifically related to COVID-19 on general distress the following day, allowing for longitudinal analysis of proximal effects. The research included data collected early in the pandemic when stressors were most novel, and included a large sample size with >80,000 assessments across 546 participants.

### Future Directions

Additional research using more comprehensive assessments of media exposure is needed to examine the differential effects of incidental exposure to news about COVID-19 (eg, seeing distressing headlines posted on social media) compared to intentional engagement. Collecting more fine-grained data (ie, momentary assessments) of media exposure may illuminate a proximal temporal precedent between worry and news exposure, allowing researchers to examine the potentially reciprocal relationship between anxious symptoms and information gathering through news engagement. To promote preventive behaviors and knowledge about the pandemic without impacting mental health, future research should also examine whether certain types of information and news delivery differentially contribute to optimism, worry, and hopelessness.

## Appendix

Multimedia Appendix 1 Temporal multilevel mediation model with person means.

Multimedia Appendix 2 Temporal multilevel mediation models with next-day worst-point outcomes. Note: the vertical dashed line indicates the break between consecutive days of data (ie, boxes shown in the same color represent data collected on the same day). Models show the effect of variables at time T (outlined in black) on variables the following day (T+1; outlined in blue).

## Abbreviations

CFI: comparative fit index
EMA: ecological momentary assessment
ICC: intraclass correlation
RMSEA: root-mean-square error of approximation
