# Supplementary material for: The Mental Health Impact of Daily News Exposure During the COVID-19 Pandemic: Ecological Momentary Assessment Study
Source: JMIR Ment Health. 2022 May 25;9(5):e36966. doi: 10.2196/36966 (PMC9135112; doi:10.2196/36966)
Supplement: Multimedia Appendix 1 [file mental_v9i5e36966_app1.docx]

**Table S1. Temporal multilevel mediation model with person-means.**

| Regressions | *β* | *SE* | *p* |
| --- | --- | --- | --- |
| **Level 1 (within-person)**  COVID-news exposure **->** Next-day hopelessness | -0.011 | 0.010 | 0.271 |
| COVID-news exposure **->** Next-day general worry  COVID-news exposure **->** Worry about COVID-19 | 0.047  0.111 | 0.013  0.015 | <0.001  <0.001 |
| COVID-news exposure **->** Optimism about COVID-19 | 0.029 | 0.011 | 0.007 |
| Worry about COVID-19 **->**  Next-day hopelessness  Worry about COVID-19 **->**  Next-day general worry  Optimism about COVID-19 **->**  Next-day hopelessness  Optimism about COVID-19 **->**  Next-day general worry  **Level 2 (between-person)**  **Variables person-centered**  COVID-news exposure **->** Next-day hopelessness  COVID-news exposure **->** Next-day general worry  COVID-news exposure **->** Worry about COVID-19  COVID-news exposure **->** Optimism about COVID-19  Worry about COVID-19 **->**  Next-day hopelessness  Worry about COVID-19 **->**  Next-day general worry  Optimism about COVID-19 **->**  Next-day hopelessness  Optimism about COVID-19 **->**  Next-day general worry  **Indirect effects**  COVID-news exposure -> Next-day hopelessness  (Via worry about COVID-19)  COVID-news exposure -> Next-day general worry  (Via worry about COVID-19)  COVID-news exposure -> Worry about COVID-19  (Via optimism about COVID-19)  COVID-news exposure -> Optimism about COVID-19  (Via optimism about COVID-19)  Total | 0.105  0.233  -0.016  -0.069  *β*  -0.079  0.111  0.685  0.021  0.213  0.211  -0.331  -0.372  *β*  0.012***  0.026***  0.000  -0.002  0.071*** | 0.015  0.019  0.014  0.018  *SE*  0.083  0.092  0.044  0.035  0.072  0.080  0.090  0.099  *LLCI*  0.008  0.018  -0.002  -0.004  0.031 | <0.001  <0.001  0.268  <0.001  *p*  0.342  0.228  <0.001  0.546  0.003  0.009  <0.001  <0.001  *UCLI*  0.016  0.034  0.000  0.000  0.111 |

_______________________________________________________

***Note:*** *Predictor and mediator variables were lagged to predict next-day outcomes (i.e., COVID-19 media exposure, COVID-19 worry, and COVID-19 optimism at time T predicted hopelessness and general worry at time T+1). SE = standard error, LLCI = lower level of the 95% confidence interval, ULCI = upper level of the 95% confidence interval*

**p < .05, **p < .01, ***p < .001*
