# Supplementary figures and images for: The Mental Health Impact of Daily News Exposure During the COVID-19 Pandemic: Ecological Momentary Assessment Study
Source: JMIR Ment Health. 2022 May 25;9(5):e36966. doi: 10.2196/36966 (PMC9135112; doi:10.2196/36966)

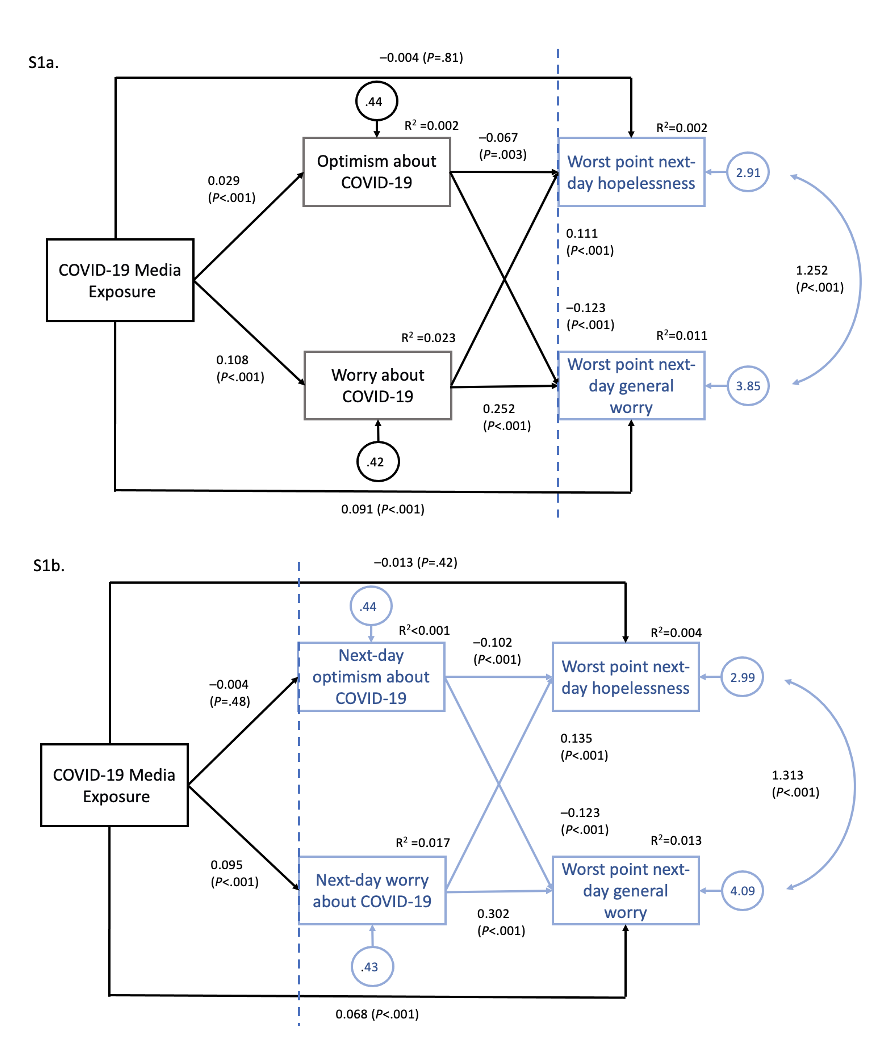

Supplement: Multimedia Appendix 2 [file mental_v9i5e36966_app3.png]
